# 

**Published:** 1958-10

**Authors:** 


MEDICAL JOURNAL OF THE SOUTH-WEST

*(Incorporating the Bristol Medico-Chirurgical Journal)*

Established 1883

VOL. 73 (iv) October 1958 NO. 270

### *Contents*

| | | Page |
|---|---|---|
| THE INVESTIGATION OF PERSISTENT DIARRHOEA | *Dr. John Naish* | 87 |
| MODERN DEVELOPMENTS IN PERIPHERAL VASCULAR SURGERY | *Dr. R. E. Horton* | 92 |
| PERINATAL MORTALITY . . . | *Dr. Thomas E. Oppé* | 99 |
| AN EXHIBITION OF IMPORTANT ANATOMICAL BOOKS | *Mr. A. E. S. Roberts* | 104 |
| HEREDITARY TELANGIECTASIA : HODGKIN'S DISEASE | *Clinical Pathological Conference* | 108 |
| NEWS FROM SOCIETIES . . . . . . | | 113 |
| NOTES AND NEWS . . . . . . . | | 116 |
| APPOINTMENTS . . . . . . . . | | 117 |

*PUBLISHED QUARTERLY*

*ANNUAL SUBSCRIPTION 16/- POST FREE*

PUBLISHED UNDER THE AUSPICES OF THE
BRISTOL MEDICO-CHIRURGICAL SOCIETY

### *Editorial Committee*

Dr. J. E. CATES, Department of Medicine, Bristol Royal Infirmary. *Editor.*

Dr. N. J. BROWN, Southmead Hospital, Bristol. *Assistant Editor.*

Dr. J. MACRAE, Ham Green Hospital.

Dr. R. C. WOFINDEN, Central Clinic, Bristol.

Mr. A. E. S. ROBERTS, Medical Library, University of Bristol. *Secretary.*

### *Local Correspondents*

*Bath* . . Mr. A. D. BATEMAN, 3 The Circus, Bath.

*Exeter* . . Dr. L. N. JACKSON, Mount Jocelyn, Crediton, Devon.

*Gloucester* . Dr. R. F. JARRETT, 9 College Green, Gloucester.

*Plymouth* . Dr. C. R. CROFT, Lexden, Hartley Av., Plymouth.

*Taunton* . Dr. M. McALLEY, 1 The Crescent, Taunton.

*Torquay* . Dr. A. ROBINSON THOMAS, 20 Devon Square, Newton Abbot, Devon.

*Truro* . . Dr. F. D. M. HOCKING, St. Andrews, Perranporth, Cornwall.

*Yeovil* . . Mr. J. A. VERE NICOLL, Knapp House, Yeovil, Somerset.

### NOTICE TO CONTRIBUTORS

Original articles are invited, provided they have not been published elsewhere. Notes of forthcoming events, meetings held, degrees conferred, staff appointments, and other local news are welcomed. The editorial committee reserves the right to make literary corrections.

Illustrations should always be supplied ready for reproduction. All re-touchings or other operations required will be charged to the author. Line drawings should be drawn in Indian ink. We regret that we must ask authors to pay for making blocks, if this is necessary.

J. W. ARROWSMITH LTD., WINTERSTOKE ROAD,
BRISTOL.

### *Notes and News*

### BRISTOL MEDICAL DRAMATIC CLUB

The next production will be " Dr. Angelus " by James Bridie at the Y.W.C.A., 30th Oct.–1st Nov. Tickets (5/– and 3/6) may be obtained from Dr. P. B. Bailey, Whitchurch. Cast: James Hope-Scott, Eve Borland, Roger Carr, Joy Loxton, John Naish, Kenneth Malcolmson, Anne Malone, Julian Benson, Christopher Hallett.

### WESSEX RAHERE CLUB

*Secretary:* Mr. A. Daunt Bateman, 11 The Circus, Bath

1st Nov.: **Dinner** at Grand Spa Hotel, Bristol. G. Bodley Scott, Esq., D.S.O., M.R.C.S., L.R.C.P., will be the guest of honour.

Membership of the Club is open, without subscription, to all Barts. Graduates practising or resident in the West Country.

### PHYSIOTHERAPISTS' CONGRESS

This year the Chartered Society of Physiotherapy held its Annual Congress in Bristol. The programme extended over three days, 11th–13th September and much of the intellectual fare was provided by local talent.

The lectures, which were delivered to an appreciative audience of over 400 included one by Mr. R. H. R. Belsey about the dangers and prevention of lung cancer, from which a quotation found its way into *The Observer's* "Sayings of the Week". Mr. H. K. Lucas gave a stimulating account of modern management of the handicaps due to poliomyelitis and dwelt in detail on aids and advice for the paralysed housewife. Mr. K. H. Pridie discussed the surgical repair of arthritic knees—a stiff painless knee is more useful than a bent or painful one—and a physiotherapist should educate the patient how to adapt himself to this compromise.

Dr. R. C. Wolfinden suggested tackling the ever-mounting toll of coronary artery disease by diet and exercise. He also advocated wider use of home care: patients preferred it, money would be saved, and hospital beds used to better purpose.

The entertainments included visits to various Medical show-pieces with a tantalizing counter-attraction at the same time of a visit to the Wine Cellars of Messrs. Harvey. There was either a reception or a dinner each evening. In after-dinner speeches Mr. A. L. Eyre-Brook talked eruditely about the history of Bristol, and Mr. Norman Capener applauded the value of actual massage itself and enjoined doctors and physiotherapists to co-operate closely for the benefit of the patient.

### PRIZES

*L. E. Attenborough Silver Gilt Medal*: B. L. Dahl.
*L. E. Attenborough Bronze Medal*: G. S. Jones.
*Tibbits Memorial Prize*: M. Nicholas.
*David Rayner Prize in Gynaecology*: Alison J. McConnell.
*Green-Armytage Prize in Obstetrics and Gynaecology*: R. A. Sharpe.
*Gold Medal in Child Health and Paediatrics*: Susi G. Leyton.

### EXAMINATION RESULTS

*M.R.C.S., L.R.C.P.*: T. O. Dada, Glenys R. Packer, E. O. Olurin.

### *Appointments*

### *UNITED BRISTOL HOSPITALS*

| *Name* | *Appointment* | *Formerly* |
|---|---|---|
| VOWLES, K. D. J., M.B.(BRISTOL), F.R.C.S. | Lecturer in Surgery, University of Bristol. Hon. Senior Registrar. | |
| WRIGHT, D. H., B.SC., M.B., CH.B.(BRISTOL) | Research Assistant, Registrar grade. | |

H. Summers, M.B.(Manch.), M.SC.(Bristol) has been appointed Assistant M.O. Mid-Cheshire area.

R. J. K. Tallack, M.B.(Bristol), D.P.H., has been appointed Assistant M.O.H. and Schoo M.O., Westmoreland.

D. M. M. Thomson, M.B.(Bristol), has been appointed M.O., Uganda.

### *SOUTH WESTERN REGIONAL HOSPITAL BOARD*

*July–August*, 1958

### *BRISTOL*

| *Name* | *Appointment* | *Formerly* |
|---|---|---|
| PAGE, F. T., M.D.(LOND.), M.R.C.P.(LOND.). | Consultant Physician with an interest in Neurology to the Bristol Clinical Area. | Senior Medical Registrar, Central Middlesex Hospital. |
| LESLIE, R. F., M.B., B.S.(LOND.). | Registrar to Bristol Chest Clinic and associated hospitals. | House Physician, Tehidy Hospital, Camborne. |
| MATTS, S. G. FLAVEL, M.B., CH.B.(B'HAM), M.R.C.P.(EDIN.). | Medical Registrar, Southmead Hospital. | Senior R.M.O. and Medical Asst. to Neurosurgical and Thoracic Surgical Depts., Frenchay Hospital. |
| FLOOD, R. A., M.B., CH.B.(ST. ANDREWS). | Registrar, Bristol Mental Hospital Group. | Senior House Officer, Barrow Hospital. |
| SPEAR, F. G., M.B., CH.B. (BRISTOL), D.P.M. (CONJOINT) | Registrar, Bristol Mental Hospital Group. | Medical Officer, Royal Air Force. |
| NABAR, B. V., M.S. (BOMBAY), F.R.C.S. (EDIN.). | Surgical Registrar, Southmead Hospital. | Surgical Registrar, Oldham and District General Hospital. |

### NORTH GLOUCESTERSHIRE

| Name | Appoinement | Formerly |
|---|---|---|
| CHAKRABARTI, R., M.B. (CALCUTTA). | Medical Registrar, Gloucestershire Royal Hospital, Gloucester. | Medical Registrar, Grimsby General Hospital. |

### BATH

| Name | Appointment | Formerly |
|---|---|---|
| WILLIS, A. J. P., M.B., B.S. (LOND.). | Medical Registrar, Bath Group of Hospitals. | S.H.O. in Pathology, Mile End Hospital, London. |

### DEVON AND CORNWALL

| Name | Appointment | Formerly |
|---|---|---|
| PICKERING-PICK, M. E. M.B., B.S. (LOND), D.A. | Anaesthetic Registrar, South Devon and East Cornwall Hospital, Plymouth. | S.H.O. Anaesthetist, South Devon and East Cornwall Hospital, Plymouth. |
| MANSFIELD, G. C., M.B., CH.B. (GLASGOW), D.R.C.O.G., D.A. | G.P. Anaesthetist, South Hams Hospital, Kingsbridge. | Is in general practice in Totnes and G.P. Anaesthetist at Paignton and District Hospital. |
| BAILEY, L. J., L.D.S., R.C.S. | S.H.D.O., Newton Abbot Hospital. | In practice in Newton Abbot. |
| DONOVAN, J. F., M.R.C.S., L.R.C.P., D.P.M. | Medical Superintendent, St. Lawrence's Hospital, Bodmin. | Consultant Psychiatrist and Physician Supt., Broadgate Hospital, Beverley, Yorks. |
| DHANDA, S. P., M.B., B.S. (MADRAS). | Surgical Registrar, South Devon and East Cornwall Hospital, Plymouth. | Surgical Registrar, Workington Infirmary. |
| LLEWELYN, D. M., M.B., B.S. (SYDNEY). | Surgical Registrar, Royal Cornwall Infirmary, Truro. | Orthopaedic Registrar, Hammersmith Hospital. |

### 1958 Index of Vol. 73

Accidental Poisoning with the Antihistamine Drug "Histantin", 45

An Exhibition of Important Anatomical Books.—A. E. S. Roberts, 104

Bradbeer, J. W.—Carcinoma of the Thyroid, 15

Carcinoma of the Thyroid.—J. W. Bradbeer, 15

Clinical Pathological Conferences:
—Accidental Poisoning with the Antihistamine Drug "Histantin", 45
—Hereditary Telangiectasia: Hodgkin's Disease, 108
—Kyphoscoliosis with Pulmonary Hypertension, 22
—Meningococcal Septicaemia with Bilateral Adrenal Haemorrhage (Waterhouse-Friderichsen Syndrome), 78

Darling, Prof. A. I.—Pain Related to the Teeth and Jaws, 36

Diseases in Search of Viruses.—Prof. C. H. Stuart-Harris, 29

Gillespie, W. A.—Hospital Cross-Infection 56

Hereditary Telangiectasia: Hodgkin's Disease, 108

Horton, R. E.—Some Modern Developments in Peripheral Vascular Surgery, 92

Hospital Cross-Infection.—W. A. Gillespie, 56

Houghton, E. A. W.—Supportive Treatment of Acute Cor Pulmonale due to Massive Pulmonary Embolism, 41

Kyphoscoliosis with Pulmonary Hypertension, 22

Medical Education, Some Thoughts of.—C. Bruce Perry, 3

Meningococcal Septicaemia with Bilateral Adrenal Haemorrhage (Waterhouse-Friderichsen Syndrome), 78

Naish, John.—The Investigation of Persistent Diarrhoea, 87

On Growing Old.—J. H. Sheldon, 69

Oppé, Thomas E.—Perinatal Mortality, 99

Pain Related to the Teeth and Jaws.—Prof. A. I. Darling, 36

Perinatal Mortality.—Thomas E. Oppé, 99

Perry, C. Bruce.—Some Thoughts of Medical Education, 3

Roberts, A. E. S.—An Exhibition of Important Anatomical Books, 104

Roberts, A. E. S.—The Medical Library of the University of Bristol, 12

Sheldon, J. H.—On Growing Old, 69

Some Modern Developments in Peripheral Vascular Surgery.—R. E. Horton, 92

Some Thoughts of Medical Education.—C. Bruce Perry, 3.

Stuart-Harris, Prof. C. H.—Diseases in Search of Viruses, 29

Supportive Treatment of Acute Cor Pulmonale due to Massive Pulmonary Embolism.—E. A. W. Houghton, 41

The Investigation of Persistent Diarrhoea.—John Naish, 87

The Medical Library of the University of Bristol.—A. E. S. Roberts, 12

